# MicroRNA expression profiling of the fifth-instar posterior silk gland of *Bombyx mori*

**DOI:** 10.1186/1471-2164-15-410

**Published:** 2014-05-29

**Authors:** Jisheng Li, Yimei Cai, Lupeng Ye, Shaohua Wang, Jiaqian Che, Zhengying You, Jun Yu, Boxiong Zhong

**Affiliations:** College of Animal Sciences, Zhejiang University, Hangzhou, Hangzhou, 310058 P.R. China; Institute of Sericulture, Chengde Medical University, Chengde, 067000 P.R. China; Key Laboratory of Genome Sciences and Information, Beijing Institute of Genomics, Chinese Academy of Sciences, Beijing, 100029 China

**Keywords:** MicroRNA, Silkworm, Posterior silk gland, Target gene

## Abstract

**Background:**

The growth and development of the posterior silk gland and the biosynthesis of the silk core protein at the fifth larval instar stage of *Bombyx mori* are of paramount importance for silk production.

**Results:**

Here, aided by next-generation sequencing and microarry assay, we profile 1,229 microRNAs (miRNAs), including 728 novel miRNAs and 110 miRNA/miRNA* duplexes, of the posterior silk gland at the fifth larval instar. Target gene prediction yields 14,222 unique target genes from 1,195 miRNAs. Functional categorization classifies the targets into complex pathways that include both cellular and metabolic processes, especially protein synthesis and processing.

**Conclusion:**

The enrichment of target genes in the ribosome-related pathway indicates that miRNAs may directly regulate translation. Our findings pave a way for further functional elucidation of these miRNAs and their targets in silk production.

**Electronic supplementary material:**

The online version of this article (doi:10.1186/1471-2164-15-410) contains supplementary material, which is available to authorized users.

## Background

The silkworm *Bombyx mori* is the most economically important holometabolous lepidopteran and has recently became an experimental model for molecular entomology [1, 2]. Its silk gland is the most efficient protein synthesis machine among all organisms, which makes silkworm a desirable model for studying its mechanism. As the largest and most important part of the silk gland, the posterior compartment is most attractive since it synthesizes the silk core protein that determines the quality of silk cocoons. A recent proteomic study, using two-dimensional gel electrophoresis (2-DE) coupled with matrix-assisted laser desorption/ionization–time-of-flight mass spectrometry (MALDI-TOF MS), has identified 93 major proteins in the silk gland, of which there are several phosphorylated fibroin L-chain and P25 isoforms [3]. The posterior silk gland of the fifth instar has been further surveyed systematically for the understanding of molecular basis and regulatory mechanism of the posterior silk gland development and fibroin synthesis [4]. A recent transcriptomic survey has revealed a total of 10,393 active genes differentially expressed in multiple silkworm tissues on the third day of the fifth-instar larva, of which 412 and 109 are up-regulated in the anterior-middle and the posterior silk glands, respectively [5]. These findings all provide basic data for studying the growth of the posterior silk gland and fibroin synthesis. However, microRNAs (miRNAs)-based study has not been done for the silk gland and its developing and functionally important compartments albeit justifiable for necessity [6–8].

As a large family of endogenous small non-coding RNAs, miRNAs are common regulatory RNAs of eukaryotic organisms and play important roles in a wide range of biological processes under physiological and pathological conditions [9–14]. Despite lack of empirical data, computational approaches have made initial contributions to miRNA study in *B. mori*[15–18], followed by next-generation sequencing efforts that profiled miRNAs for different developmental stages and tissues [19–22]. Nevertheless, specifically focused study is still necessary since the expression of miRNAs is largely temporal-spatial [22–25].

Here, we report our miRNA profiling of the fifth-instar posterior silk gland, using next-generation sequencing and microarray technologies. We show that 728 out of 1,229 miRNAs are novel and 430 of the total are identified in the third day of the posterior silk gland development. Our GO (Gene Ontology)-based pathway assignment provides the first comprehensive categorization of *B. mori* miRNAs in the posterior silk gland.

## Results and discussion

### Next-generation sequencing and data processing

**Figure 1:**
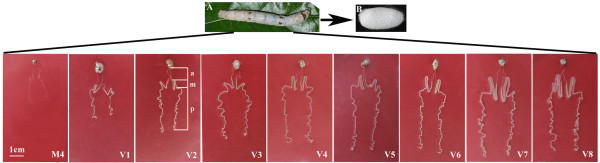
**The silk gland from the fourth molting to the fifth instar day 8.** The fifth-instar silkworm larvae **(A)** and cocoon **(B)**. M4 to V8 represent nine consecutive days of the silk glands developmental stages from the fourth molting larva to the fifth-instar larvae (V1 to V8). a, the anterior silk gland; m, the region of the middle silk gland; and p, the posterior silk gland.

Rapid growth of the silk gland occurs at the fifth instar stage, and the gland is comprised of three distinct compartments: the anterior, the middle, and the posterior glands (Figure 1). Compared with the other two parts, the anterior gland, albeit smaller, serves as a duct to transport (spinning) silk proteins that form the cocoon. The middle gland produces considerable quantities of sericins and the longest posterior gland grows rapidly, synthesizing a series of proteins including fibroin heavy and light chains plus fibroin P25 by exclusively ~500 posterior gland cells of the fifth instar larva. As far as the biosynthesis of fibroin is concerned, the fifth instar stage can also be partitioned into two periods: the rapid formation and the massive secretion [26]. The third day of the fifth instar (V3) completes a division during larval development and rapid cell growth occurs at this period of time. Based on data from genome-scale expression profiling of the posterior silk gland, it has been concluded that gene expression profile from the fourth instar molting to the fifth instar day 8 before spinning forms two clusters that is divided at V3 from the fourth molting to wandering periods [4]. A large amount of genes encoding the fibroin light chain, fibrohexamerin P25, transcription factors, structural proteins, glucose and other sugar transporters and proteins that aid in hormone signal transduction are up-regulated in the posterior silk gland from V1 to V5, and are slightly down-regulated at the wandering stage [4, 5]. Therefore, changes of gene expression at the fifth instar may be responsible for growth and development of the posterior silk gland, especially various miRNAs that play regulatory roles in post-transcriptional control [27].

The raw and processed data of all samples have been deposited in NCBI’s Gene Expression Omnibus (GEO) [28] under accession number GSE 56380. From 93.2 million processed reads ranging from 18 ~ 30 nt in length (Table 1, Additional file 1: Figure S1), we first examined the length distribution of small RNAs from ten libraries and found two extremely high peaks in most libraries; one was around 20 nt and the other around 28 nt (Additional file 2: Figure S2). This result is consistent with the previous reports, where the first peak was proposed to represent miRNAs and the other was interpreted as longer piRNA-like small RNAs [21, 22, 29, 30]. We subsequently categorized non-coding small RNAs and defined them according to Rfam database 10.0. The individual expression level of small RNAs is very similar across the 10 libraries (Additional file 3: Table S1). We found that rRNAs and tRNAs were the majority of all non-coding RNA categories, as they are accounted for the most components of protein synthesis.

**Table 1:** **Data summary of sequenced small RNAs based on DSAP**

	Q1	Q2	B1	B2	QB1	QB2	BQ1	BQ2	R1	J1
**Total reads**	18707473	18633295	18556476	18499427	17947940	16942708	17449851	17905942	10508573	12079631
**Cleaned sequence tags**	901684	674947	810182	622202	633421	617616	857114	617471	1162861	816340
**Reads in cleaned sequence tags**	10044506	10693830	10790311	9085018	7461204	8113977	9405884	9159736	8614699	9822808
**% reliable reads** ^**#**^	53.69	57.39	58.15	49.11	41.57	47.89	53.90	51.15	81.98	81.32
**Unique Sequence Clusters** (**USC**)	901684	674947	810182	622202	633421	617616	857114	617471	1162861	816340
**Matched ncRNA in Rfam**	690	608	673	596	642	569	701	602	739	693
**USC matched to Rfam**	134580	97673	122849	105997	89704	92104	127959	101047	154898	145107
**Reads matched to Rfam**	2729256	2440852	2908825	2573860	1644409	2172466	2133486	2565135	1108916	1386447
**% reads matched to Rfam**	27.17	22.82	26.96	28.33	22.04	26.77	22.68	28	12.87	14.11
**Matched miRNAs in miRBase**	181	170	182	157	181	150	202	168	239	220
**USC matched to miRBase**	1411	1167	1216	927	1174	817	1643	1016	2540	2089
**Reads matched to miRBase**	406154	744285	540458	277369	486835	339576	544337	310026	1486269	1576622
**% reads matched to miRNAs**	4.04	6.96	5.01	3.05	6.52	4.19	5.79	3.38	17.25	16.05
**USC Unmatched reads**	765693	576107	686117	515278	542543	524695	727512	515408	1005423	669144

Note: #Percentage of reliable reads = (Number of reads in cleaned sequence tags/Number of total reads)*100.

### Known and novel miRNAs based on sequence data

**Figure 2:**
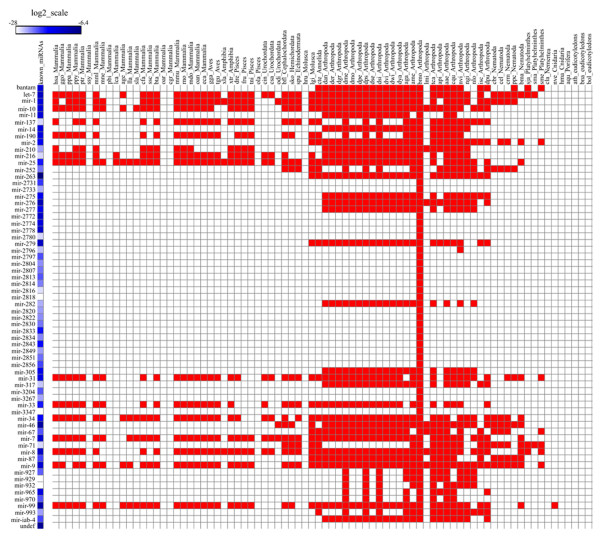
**Cross-** **species analysis of known miRNA families.** All known miRNAs are classified into 66 miRNA families and one undefined group. The known miRNAs are distributed over 68 species; among them 40 families are widely conserved in insects and 26 families are unique to *B. mori*.

After the removal of larger RNAs, we mapped the remaining reads (18–30 nt) to miRBase 16.0 [31] using the deep-sequencing small RNA analysis pipeline (DSAP). DSAP is a fast web server specially designed to analyze known miRNAs generated from the Illumina sequencing platform and yields satisfactory results [32, 33]. Our effort yielded 304 known miRNAs (Additional file 4: Table S2), accounted for a large proportion of miRBase 16.0 (http://www.mirbase.org/cgi-bin/browse.pl), which are 20–27 nt in size and have the highest abundance (71.38%) in a range of 21–23 nt. We grouped them into 66 miRNA families except for some undefined miRNAs (Additional file 4: Table S2 and Additional file 5: Figure S3). Based on cross-species analysis, these known miRNAs are shared by ~68 species. Among them, 40 families are widely conserved in insects and 26 families are unique to *B. mori*. Moreover, 25 families are distributed among 14 classes/phyla including both invertebrates and vertebrates (Figure 2 and Additional file 6: Figure S4).

The read count for different miRNA is rather variable (Additional file 4: Table S2). For instance, the number of reads for bmo-miR-263a is extremely high as compared to other miRNAs in all libraries; it may play a very important role in the posterior gland development and the result is in agreement with a previous report [20]. We also found 50 pairs of miRNA/miRNA* duplexes, in addition to 24 miRNA*s without the corresponding miRNAs. Although most miRNAs are more abundant than their corresponding miRNA*s, there are exceptional cases, where bmo-miR-10, bmo-miR-276, bmo-miR-305, bmo-miR-33, and bmo-miR-34 are less abundant than their miRNA* counterparts. Similar findings have also been reported in other deep sequencing experiments and are suspected to be a result of incorrect annotations in miRBase [34–36].

Having filtered the known non-coding RNAs, we predicted novel candidate miRNAs using the mireap package [37] and classified 1,427 candidate miRNAs (Additional file 7: Table S3). Given the fact that there are many random inverted repeats (termed pseudo-hairpins) in eukaryotic genomes and they can also fold into dysfunctional hairpins and undistinguished sequences, we took extra cautions to classify non-conserved miRNAs. We used mirident classifier to identify the miRNA candidates, which has been reported to achieves 99.2% specificity and 97.6% sensitivity on a human test data set [38]. We also evaluated two other SVM-based prediction programs, Triplet-SVM and PmirP, together with mirident and using miRbase datasets that include data from 24 insect species [38–40]. Mirident classifier gave rise to better results for insect pre-miRNA identification in our own hands [41]. Using mirident classifier, we obtained 613 novel miRNAs, corresponding to 590 unique sequences after filtering pseudo-pre-miRNAs (Additional file 7: Table S3).

### Microarray-based miRNA profiling

Since the third day of fifth-instar larva (V3) is a key time point for silk synthesis and rapid cell growth, we evaluated its miRNA expression profile using 3,077 custom-designed probes (Additional file 1: Figure S1 and Additional file 8: Table S4) that are classified into four group: (1) 1,006 known miRNAs from miRBase, which consist of miRNAs from several species, including silkworm (559 probes) and 10 flies (447 probes); (2) 1,427 predicted novel miRNAs; (3) 425 probes based on data from four publications [17, 19, 22, 42]; and (4) 219 control sequences. To ensure reproducibility, we double-gridded 841 sequences with read coverage of >5 from 1,427 custom-designed probes for each chip.

We used 16 chips (probe sets) for the study and normalized the data in log2 transformation. Both technical (all R^2^ > 0.97) and biological repeats (all R^2^ > 0.8) showed consistent results (Additional file 9: Figure S5 and Additional file 10: Table S5). As miRNA-based microarray experiments in general are reproducible [43–45], we readily identified 430 mature miRNAs. Among them, 239 are previously known and the remaining 191 include 19 conserved in Drosophila and 172 novel ones (Additional file 11: Table S6). Of the 239 known miRNAs, 187 are from a thorough collection from literature search and the remaining 52 are directly from the miRBase. These miRNAs showed different expression patterns among the samples and did not exhibit any obvious correlation to information sources. For instance, bmo-bantam, bmo-miR-12, bmo-miR-263a, bmo-miR-263b, bmo-miR-278, and bmo-miR-8 are both literature-based and database collections but the miRBase-collected miRNAs showed stronger signals. The contrary results were found among bmo-let-7, bmo-miR-1, bmo-miR-100, bmo-miR-124, bmo-miR-137, bmo-miR-14, bmo-miR-252, bmo-miR-275, bmo-miR-305, bmo-miR-307, bmo-miR-34, and bmo-miR-279c, where the literature-based collections showed higher expression levels (Additional file 12: Table S7). We inferred that this non-uniformity might be a result of different technical platforms. One discussion point on this study is the validation rate: only 172 novel miRNAs (~14%) from the sequencing data are confirmed in the microarray experiment. The reasons for such low confirmation are multifold. First, 430 mature miRNAs are detected on the third day of fifth instar but not on the entire stage, where samples are collected and pooled from the fourth instar molting to the fifth instar day 8 before spinning. The false negative results for some of the miRNAs are largely due to the dilution of the time-sensitive specific miRNAs over pooling [46, 47]. Second, the marginal level of miRNA expression is pushing the detection limit so that some of the signals may not be consistently detected even when the same experiments are repeated. Third, sampling bias may be inherent from the sequencing approach, where sampling bias is obvious for low copy transcripts [27, 43].

There are 257 miRNA genes whose expression patterns have been reported to correspond to 324 loci in the *B. mori* genome [22]. After the removal of redundant sequences, we found that the two datasets shared 197 miRNAs (16 sequences showing discrepancy in their sequences were not accounted for). Among these miRNAs, 75 genes showed posterior gland expression in the current study but have not been detected in the previous study. Conversely, from 173 miRNAs identified previously in two public datasets for posterior-silk-gland expression, 37 were negative in our study. In addition, seven (bmo-miR-2846, bmo-miR-2850a, bmo-miR-2853, bmo-miR-2854, bmo-miR-2858*, bmo-miR-2858, and bmo-miR-2859) out of 21 posterior-gland specific miRNAs defined previously were not detected in our microarray experiment (Additional file 13: Table S8). These results suggest that our experiment covered most of the *B mori* miRNAs but inconsistency does exist, attributable to the difference between technical platforms.

### Target gene prediction and pathway analysis

Combining results from both deep sequencing and microarray, we identified 1,229 miRNAs expressed in the posterior silk gland in the period of the fourth-instar molting to the fifth-instar (day 8 before) spinning, and among which 728 are novel, named as bmo-miR-Pxxx-xp series (from No.1 to No.728 at Additional file 14: Table S9), and 110 are miRNA/miRNA* duplexes (Additional file 15: Table S10). We also profiled 430 miRNAs for the third day of the fifth-instar larva (Additional file 11: Table S6). We subsequently predicted potential targets using miRanda v3.3a [48] and the effort yielded 14,222 targets in the entire stage, corresponding to 1,195 miRNAs. The rest 34 miRNAs did not yield target genes due to low scores (Additional file 16: Table S11). We associated 12,675 and 12,948 target genes to 423 known and 696 novel miRNAs in the third day of the fifth instar, respectively (Additional file 17: Table S12 and Additional file 18: Table S13).

**Figure 3:**
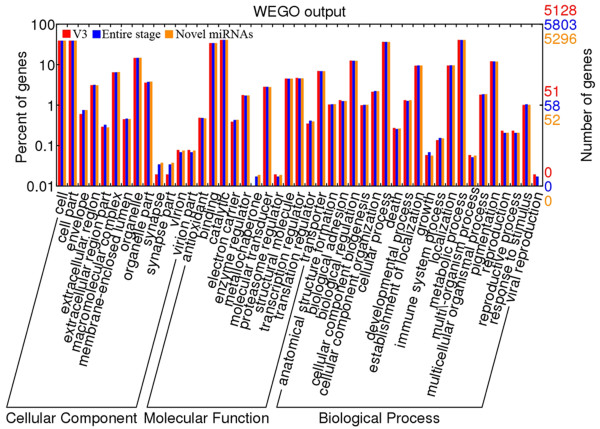
**GO analyses of the target genes predicted by miRanda in the posterior silk gland.** We analyzed three groups of miRNAs: (1) V3, 430 miRNAs detected at the third day of fifth instar; (2) Entire Stage, 1,229 miRNAs discovered at the periods from the fourth instar molting to the fifth instar day 8 before spinning, and (3) Novel, 728 miRNAs first detected in this study.

We annotated all miRNA target genes based on GO analysis and found that they are largely involved in cell, cell part, binding, catalytic, cellular process, metallochaperone, proteasome regulator, and metabolic process, as opposed to the underrepresented synapse, synapse part, and viral reproduction (Figure 3). This result suggests that miRNAs may regulate mostly the expression of structural protein genes in the posterior gland than those involved in the development of neural and immune systems. Furthermore, our GO analysis on the target genes of novel miRNAs and those detected at the third day of the fifth instar showed a similar result to that of the total miRNAs, except the absence of viral reproduction in biological process terms and metallochaperone in molecular function terms. Finally, as the major silk protein secretary organ, the posterior silk gland has an increased ribosome content [4, 49, 50], and it has been reported that ribosomal proteins are abundantly expressed in the final instar and play key roles in modulating activity of ribosome [49, 51]. Our result confirmed this observation.

**Figure 4:**
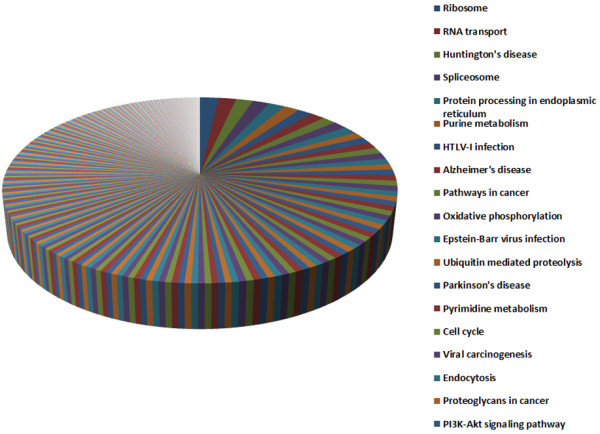
**The number of miRNA target genes mapped on pathway.** The miRNAs detected at Entire stage from the fourth instar molting to the fifth instar day 8 before spinning.

**Figure 5:**
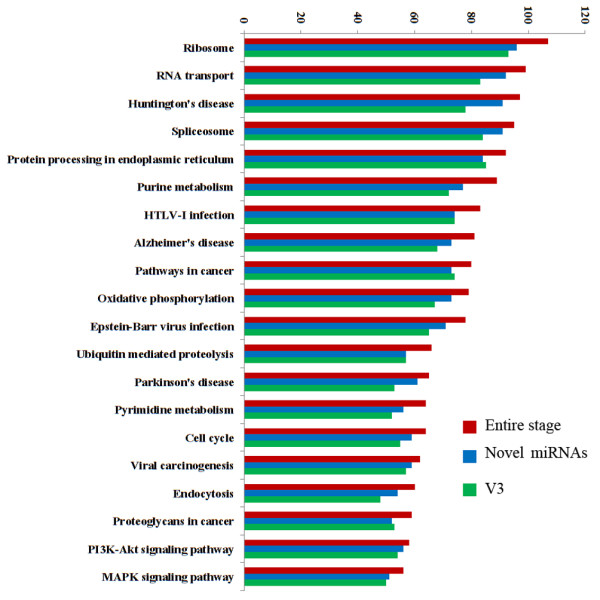
**KEGG pathways mapped based on miRNA target genes.** Entire stage, the target genes of miRNAs detected over the entire period from the fourth instar molting to the fifth instar day 8 before spinning; Novel, target genes first predicted in this study; V3, target genes detected at the third day of fifth instar.

**Figure 6:**
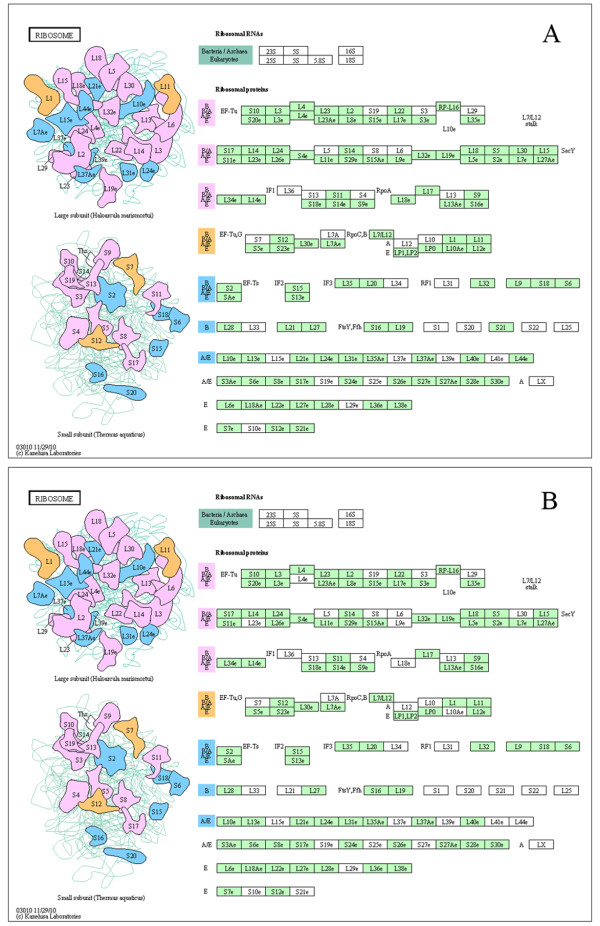
**The ribosome pathway.** miRNA target genes detected in the entire period from the fourth instar molting to the fifth instar day 8 before spinning **(A)** and target genes first detected in this study **(B)**. Mapped pathways were highlighted in green.

Based on further comparison of biological pathways among three datasets (the entire stage, the novel miRNAs, and the V3 group), we showed that 5,871 out of 14,222 predicted targets in the entire stage were involved in 302 KEGG pathways (Figure 4, Additional file 19: Table S14). The other two sets of target genes shared a similar result, where 5,400 and 5,331 target genes from the novel miRNAs and V3 group were mapped onto these pathways, respectively (Additional file 19: Table S14; Figure 5). Furthermore, there were 107 target genes mapped to the ribosome pathway (Figure 6, Additional file 20: Figure S6 and Additional file 19: Table S14) and 92 target genes involved in protein processing of endoplasmic reticulum pathway in the entire stage (Additional file 21: Figure S7 and Additional file 19: Table S14). Since translation-level regulation of ribosomal proteins is critical for fibroin synthesis [4], most of the target genes (107, 96 and 93 in the entire stage, the novel miRNA, and the V3 group, respectively) were mapped to ribosome pathway for all three datasets as compared to other pathways, and these target genes almost covered all the genes in this pathway (Figure 5, Figure 6 and Additional file 20: Figure S6). These results indicate that miRNAs expressed in the fifth instar of posterior silk gland showed strong regulatory functions on the silk protein synthesis.

Other involved pathways are also informative. First, 99 target genes are related to RNA transport pathway, and 47 target genes are mapped to RNA degradation pathway (Additional file 19: Table S14 and Additional file 22: Figure S8). Nearly 90 and 50 target genes are involved in purine and pyrimidine metabolisms, respectively (Additional file 19: Table S14, Additional file 23: Figure S9 and Additional file 24: Figure S10 ). The results indicate active regulations of transcription and nucleotide metabolism. Second, 79 target genes are found to be involved in oxidative phosphorylation pathway (Additional file 19: Table S14 and Additional file 25: Figure S11). The ATP production pathway may coordinate with nucleotide metabolic pathways for energy generation. Third, 66 and 33 target genes are related to ubiquitin mediated proteolysis and proteasome pathways, respectively (Additional file 19: Table S14 and Additional file 26: Figure S12). Since ubiquitin proteolytic system plays an important role in a broad array of basic cellular processes including regulation of cell cycle, modulation of the immune and inflammatory responses, control of signal transduction pathways, development and differentiation, and these complex processes are controlled by specific degradation of a single or a subset of proteins [52], the discovery of such a significant involvement is of importance. Fourth, we observed 64 target genes mapped to cell cycle pathway (Additional file 19: Table S14 and Additional file 27: Figure S13) which suggests that these miRNAs may be regulators of cell cycle. It has been well established that cell division only occurs during the embryonic development, and the number of cells in the posterior silk gland no longer increases throughout the larval life [53]. Finally, pathway analysis results showed highly consistency between the three datasets: the entire stage, the novel miRNAs, and the V3 group.

## Conclusion

From 10 small RNA libraries, we acquired ~93 million processed reads ranging from 18–30 nt in length, identified 1,229 miRNAs (110 miRNA/miRNA* duplexes), and profiled 430 miRNAs at the third day of the fifth instar larva. We also found 728 novel miRNAs (including 55 miRNA/miRNA* duplex and 709 Bm-specific miRNAs [54]. Our findings expanded the collection of *B. mori* miRNAs in miRBase and covered most miRNAs of the posterior silk gland. Moreover, on the discovery of target genes [51–53], we predicted 14,222 targets matching 1,195 miRNAs, which are classified into many important pathways including protein synthesis, energy supply, and cell cycle control. Our results underscore the key regulatory roles that miRNAs play in the fifth instar posterior silk gland for silk production.

## Methods

### Silkworm rearing and sample preparation

For better miRNA profiling and eliminating strain-specific effects, we selected six domesticated silkworm strains (Q, Qiufeng; B, Baiyu; QB, Qiufeng × Baiyu; and BQ, Baiyu × Qiufeng, R1, and J1) and reared them on fresh mulberry leaves under standard condition. We used three sets of samples according to genes expression cluster analysis [4]: (1) Stage 1: fourth instar molting to day 2 of fifth instar from Q, B, QB, and BQ); (2) Stage 2: fifth instar day 3 to day 8 before spinning; and (3) Entire stage: Stage 1 + 2 from R1 and J1. The samples were collected daily and dissected and stored at low temperature in 0.7% NaCl. Samples were subsequently rinsed and stored in liquid nitrogen.

### Small RNA library construction and solexa sequencing

Total RNA was extracted from the posterior silk gland with Trizol reagent (Invitrogen, Carlsbad, CA, USA) according to the manufacturer’s instructions. For miRNA-seq, total RNA of the desired size range (18–30 nt) was size-fractionated on a 15% PAGE gel and ligated with to sequence adapters (T4 RNA ligase). After amplified for 15 cycles, the products were separated on agarose gels and the RT-PCR products were sequenced on the Illumina platform (Beijing Genomics Institute or BGI, Shenzhen) [55, 56].

### Sequence analysis and microRNA prediction

Raw sequence reads of 35 nt in size were generated and unique reads of full-length small RNA sequences (≥18 nt) were analyzed with deep sequencing small RNA analysis pipeline (DSAP) (http://dsap.cgu.edu.tw/dsap.html). Unique reads matching silkworm non-coding RNA (rRNA. tRNA, sRNA, snoRNA and other non-coding RNA) deposited at the NCBI GenBank database and Rfam 10.0 were removed. The clean reads from raw dataset were matched to the known miRNA in miRBase 16.0 (http://microrna.sanger.ac.uk) to identify conserved miRNAs and annotated stem-loop sequences. After strict screening, the remaining sequences were regarded as candidate miRNAs for further analysis.

To determine potential novel miRNAs, we identified candidate miRNAs using the mireap program (http://sourceforge.net/projects/mireap), which is an algorithm developed by BGI, which can be used to identify known miRNAs and novel candidates with canonical hairpin structure and sufficiently supported by sequencing data. In the present study, mireap parameters were set to match the condition of animal miRNAs identification as follows: (1) the length range of the miRNA sequence: 20–24 nt; (2) the maximal free energy allowed for an miRNA precursor: -18 kcal/mol; (3) the minimal common base pairs between miRNA and miRNA*: 14 with no more than four bulges; and (4) the maximal asymmetry of miRNA/miRNA* duplex: 5 nt. Following miRNA prediction, secondary structures were predicted by using the Zuker algorithm that evaluates hairpin forming potential (http://rna.urmc.rochester.edu/rnastructure.html).

### Microarray analysis

To determine comprehensive miRNA expression profiles on the third day of fifth instar larvae, we collaborated with LC Bio Co. Ltd (LC sciences, USA) developed and designed miRNA probes. Considering that miRNA expression profiles may vary in different varieties and genders [46], we collected both male and female silkworms from four stains (Q, B, QB, and BQ) in duplicates. The small RNA fraction was extracted with Trizol reagent (Invitrogen, Carlsbad, CA, USA). To ensure the quality of the RNA, we used checked RNA quality and quantity with spectrophotometer and size-fractionated it using YM-100 Microcon centrifugal filter (Millipore). After adding poly-A tails, hybridization (10 μg probe) was used was carried out on a μParaflo™ microfluidic chip (Atactic Technologies) [57]. After imagine acquisition (GenePix 4000B, Molecular Device; Media Cybernetics) and background removal, we normalized the signals using a LOWESS (Locally-weighted Regression) method [58], classified the data using a hierarchical clustering method and average linkage and Euclidean distance metric, and visualized the results with TIGR MeV (Multiple Experimental Viewer; Institute for Genomic Research).

### Target gene prediction analysis

Due to lack of available 3’-utr database, we first estimated the unigenes from NCBI (release date: Mar 30, 2006) and considered 1 kb as a suitable length for silkworm 3’-utr. Then, according to the annotation of silkdb2.0 (http://www.silkdb.org/silkdb), 1 kb sequences after the last exon of annotated genes were selected as target gene region. Finally, we used miRanda v3.3a (http://cbio.mskcc.org/microrna_data/manual.html) to predict potential targets. The thresholds for candidate target sites were set at S ≥ 140 and ΔG < -20 kcal/mol [48].

### Gene ontology and KEGG pathways analysis

We analyzed the function of miRNA targets based on Gene Ontology through searching against InterPro and KEGG databases (http://www.genome.jp/kegg/), using InterProScan, WEGO (http://wego.genomics.org.cn/), and UniProtKB (http://pir.georgetown.edu/pirwww/search/blast.shtml).

## Authors’ information

JL: ^1^College of Animal Sciences, Zhejiang University, Hangzhou 310058, P.R. China, ^2^Institute of Sericulture, Chengde Medical University, Chengde 067000, P.R. China. YC: Key Laboratory of Genome Sciences and Information, Beijing Institute of Genomics, Chinese Academy of Sciences, Beijing, 100029, China.

## Electronic supplementary material

Additional file 1: Figure S1: Step-by-step workflow of the strategy for posterior silk gland of silkworm miRNA expression profiling analysis. (TIFF 69 KB)

Additional file 2: Figure S2: Length distribution of small RNAs from 10 libraries of posterior silk gland of silkworm from the Illumina data. (TIFF 387 KB)

Additional file 3: Table S1: Rfam expression level of ten libraries of posterior silk gland of silkworm. (XLS 125 KB)

Additional file 4: Table S2: Known miRNAs detected of posterior silk gland of silkworm. (XLS 66 KB)

Additional file 5: Figure S3: The length distribution of all known miRNAs summarized by DSAP. (TIFF 152 KB)

Additional file 6: Figure S4: Phylogenic distribution analysis of known miRNA families. Similar to Figure 3, we find 25 families are distributed in over 14 classes or phylums from invertebrates to vertebrates according to phylogenic distribution. (TIFF 2 MB)

Additional file 7: Table S3: Candidate novel miRNAs predicted by mireap program and identified by mirident classifier and miRAlign. (XLS 686 KB)

Additional file 8: Table S4: The composition of probes in microarray assay. (XLS 1 MB)

Additional file 9: Figure S5: Biological repeats correlation analysis. In order to verify the reliability of microarray assay, male (M) and female (F) silkworms of four stains were separately treated and repeated twice. Q stands for Qiufeng and B represents Baiyu, while QB and BQ are their reciprocal cross breeds. (TIFF 546 KB)

Additional file 10: Table S5: Significance testing for correlation of technical repeats by probes of repeated twice. (DOC 46 KB)

Additional file 11: Table S6: miRNAs of posterior silk glands confirmed by microarray assay. (XLS 92 KB)

Additional file 12: Table S7: Comparison miRNAs expression level with miRBase. (XLS 35 KB)

Additional file 13: Table S8: Comparison of miRNAs of the posterior silk glands with previous report. (XLS 76 KB)

Additional file 14: Table S9: Total and novel miRNAs detected in silkworm posterior silk gland. (XLS 185 KB)

Additional file 15: Table S10: All miRNA/miRNA* duplex detected in silkworm posterior silk gland. (XLS 52 KB)

Additional file 16: Table S11: Target genes of miRNAs detected at the entire stage prediction using miRanda. (XLS 3 MB)

Additional file 17: Table S12: Target genes of miRNAs detected at the third day of the fifth instar prediction using miRanda. (XLS 2 MB)

Additional file 18: Table S13: Target genes of novel miRNAs prediction using miRanda. (XLS 2 MB)

Additional file 19: Table S14: The number of posterior silk gland miRNA target genes mapped on pathway. (XLS 56 KB)

Additional file 20: Figure S6: The ribosome pathway. Target genes of the third day of the fifth instar detected miRNA. Mapped pathways were highlighted in green. (TIFF 462 KB)

Additional file 21: Figure S7: The oxidative phosphorylation pathway. (A) miRNA target genes detected in the entire period from the fourth instar molting to the fifth instar day 8 before spinning, (B) target genes first detected in this study; and (C) target genes detected in the third day of the fifth instar. Mapped pathways were highlighted in green. (TIFF 767 KB)

Additional file 22: Figure S8: The purine metabolism pathway. (A) miRNA target genes detected in the entire period from the fourth instar molting to the fifth instar day 8 before spinning, (B) target genes first detected in this study; and (C) target genes detected in the third day of the fifth instar. Mapped pathways were highlighted in green. (TIFF 1 MB)

Additional file 23: Figure S9: The pyrimidine metabolism pathway. (A) miRNA target genes detected in the entire period from the fourth instar molting to the fifth instar day 8 before spinning, (B) target genes first detected in this study; and (C) target genes detected in the third day of the fifth instar. Mapped pathways were highlighted in green. (TIFF 1 MB)

Additional file 24: Figure S10: The RNA transport pathway. (A) miRNA target genes detected in the entire period from the fourth instar molting to the fifth instar day 8 before spinning, (B) target genes first detected in this study; and (C) target genes detected in the third day of the fifth instar. Mapped pathways were highlighted in green. (TIFF 1 MB)

Additional file 25: Figure S11: The cell cycle pathway. (A) miRNA target genes detected in the entire period from the fourth instar molting to the fifth instar day 8 before spinning, (B) target genes first detected in this study; and (C) target genes detected in the third day of the fifth instar. Mapped pathways were highlighted in green. (TIFF 1 MB)

Additional file 26: Figure S12: The ubiquitin mediated proteolysis pathway. (A) miRNA target genes detected in the entire period from the fourth instar molting to the fifth instar day 8 before spinning, (B) target genes first detected in this study; and (C) target genes detected in the third day of the fifth instar. Mapped pathways were highlighted in green. (TIFF 765 KB)

Additional file 27: Figure S13: The protein processing in endoplasmic reticulum pathway. (A) miRNA target genes detected in the entire period from the fourth instar molting to the fifth instar day-8 before spinning, (B) target genes first detected in this study; and (C) target genes detected in the third day of the fifth instar. Mapped pathways were highlighted in green. (TIFF 757 KB)

## Abbreviations

miRNA: microRNA
2-DE: two-dimensional gel electrophoresis
MALDI-TOF MS: Matrix-assisted laser desorption/ionization–time-of-flight mass spectrometry
GO: Gene ontology
KEGG: Kyoto encyclopedia of genes and genomes
DSAP: Deep-sequencing small RNA analysis pipeline
Q: Qiufeng
B: Baiyu
QB: Qiufeng × Baiyu
BQ: Baiyu × Qiufeng.
