# Supplementary figures and images for: MicroRNA expression profiling of the fifth-instar posterior silk gland of Bombyx mori
Source: BMC Genomics. 2014 May 29;15(1):410. doi: 10.1186/1471-2164-15-410 (PMC4045974; doi:10.1186/1471-2164-15-410)

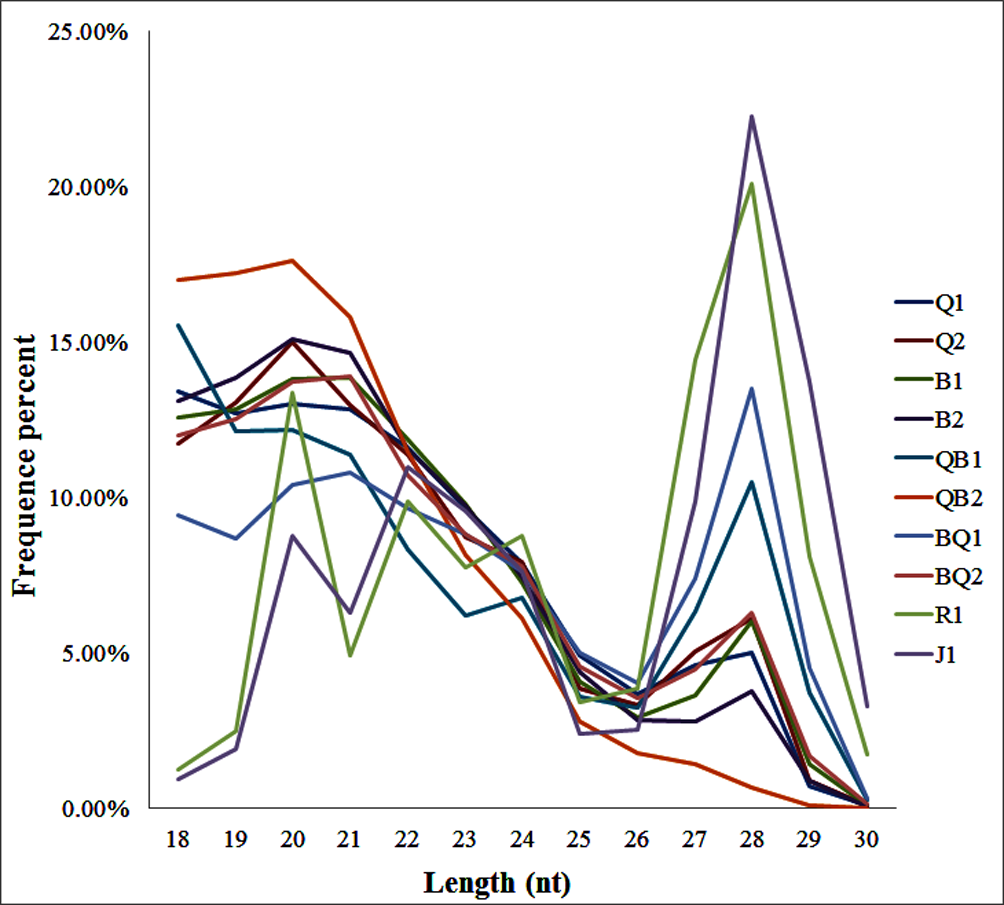

Supplement: Supplementary file 2 — Additional file 2: Figure S2: Length distribution of small RNAs from 10 libraries of posterior silk gland of silkworm from the Illumina data. (TIFF 387 KB) [file 12864_2013_6094_MOESM2_ESM.tiff]

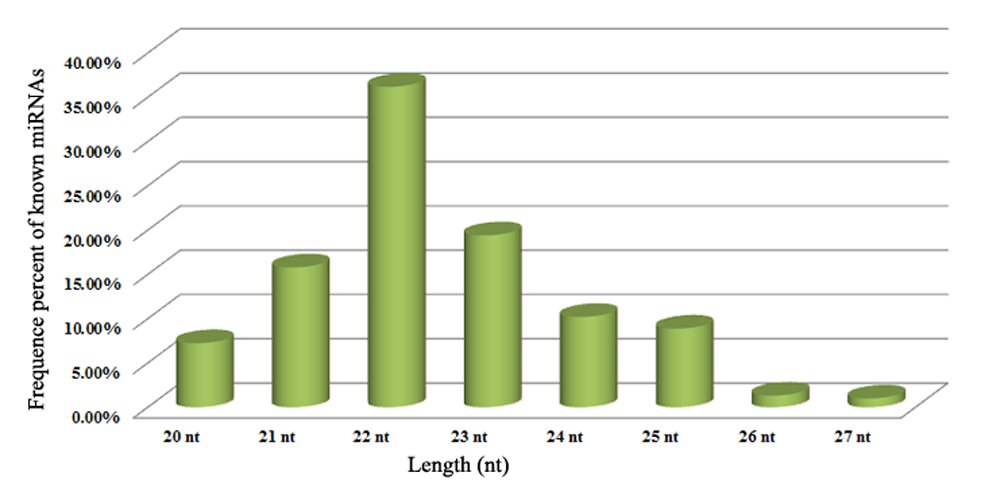

Supplement: Supplementary file 5 — Additional file 5: Figure S3: The length distribution of all known miRNAs summarized by DSAP. (TIFF 152 KB) [file 12864_2013_6094_MOESM5_ESM.tiff]

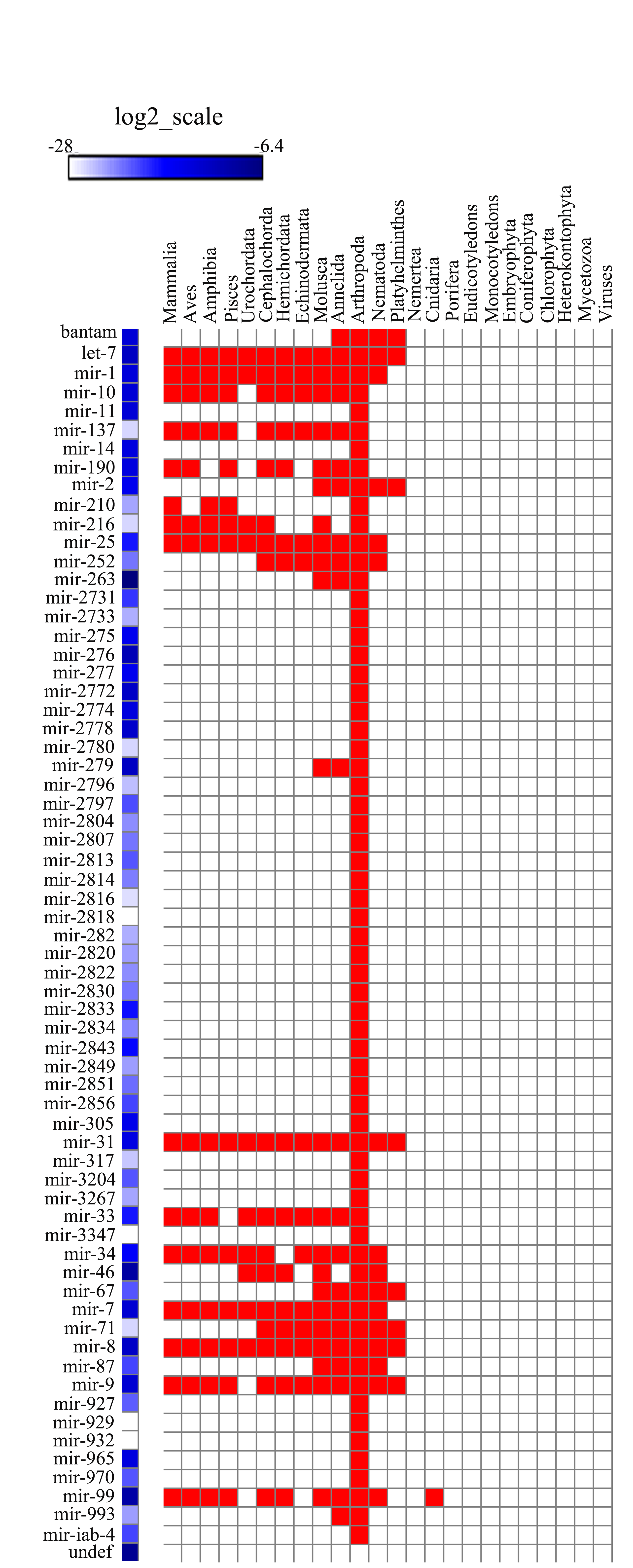

Supplement: Supplementary file 6 — Additional file 6: Figure S4: Phylogenic distribution analysis of known miRNA families. Similar to Figure 3, we find 25 families are distributed in over 14 classes or phylums from invertebrates to vertebrates according to phylogenic distribution. (TIFF 2 MB) [file 12864_2013_6094_MOESM6_ESM.tiff]

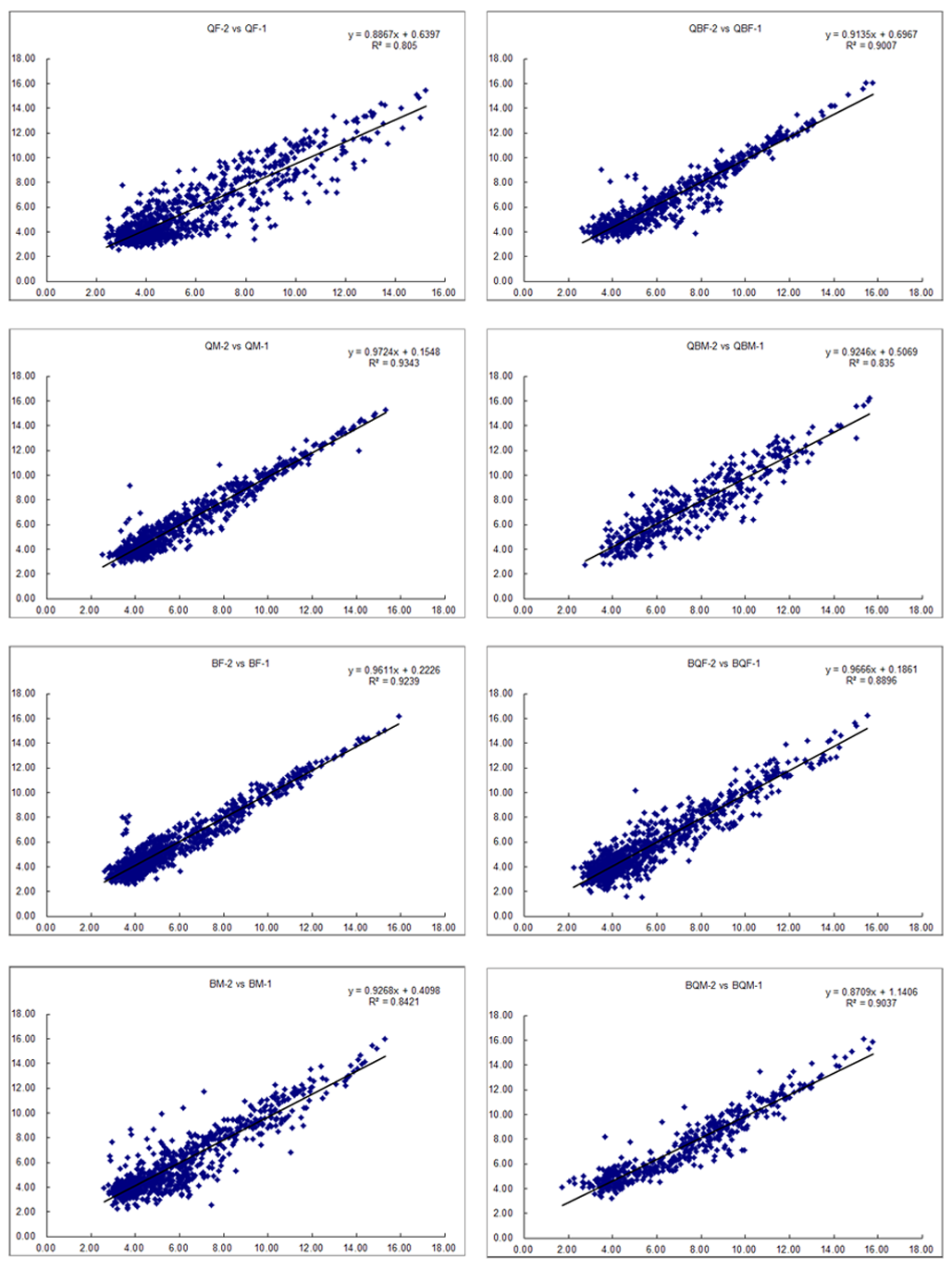

Supplement: Supplementary file 9 — Additional file 9: Figure S5: Biological repeats correlation analysis. In order to verify the reliability of microarray assay, male (M) and female (F) silkworms of four stains were separately treated and repeated twice. Q stands for Qiufeng and B represents Baiyu, while QB and BQ are their reciprocal cross breeds. (TIFF 546 KB) [file 12864_2013_6094_MOESM9_ESM.tiff]

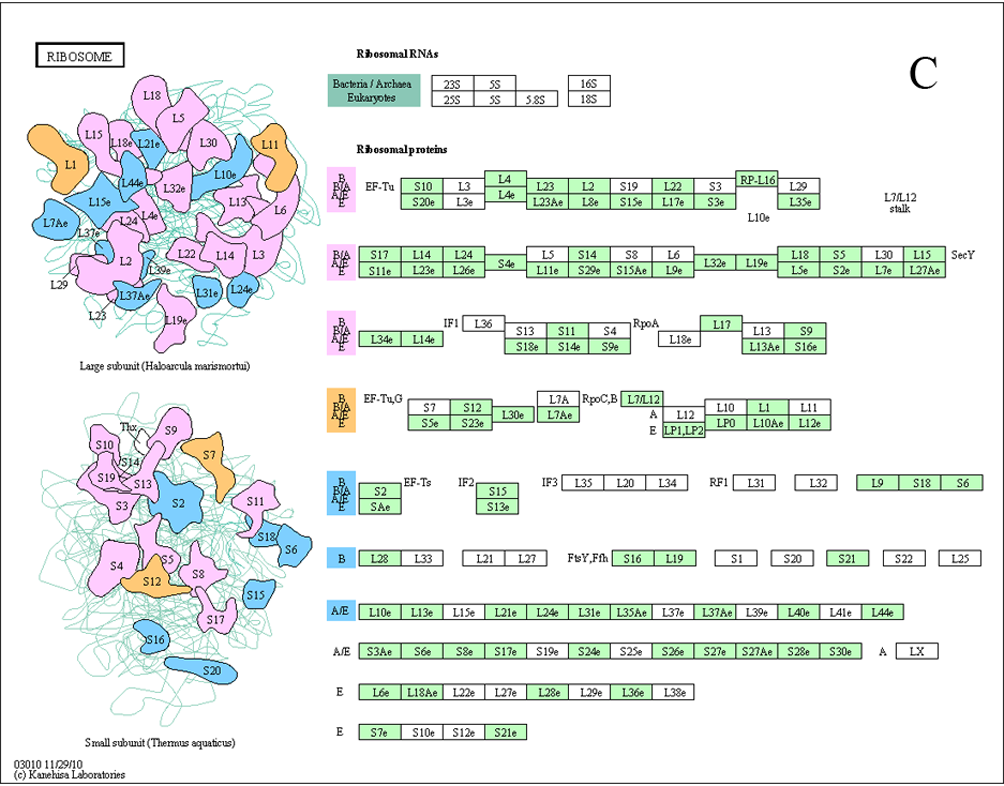

Supplement: Supplementary file 20 — Additional file 20: Figure S6: The ribosome pathway. Target genes of the third day of the fifth instar detected miRNA. Mapped pathways were highlighted in green. (TIFF 462 KB) [file 12864_2013_6094_MOESM20_ESM.tiff]

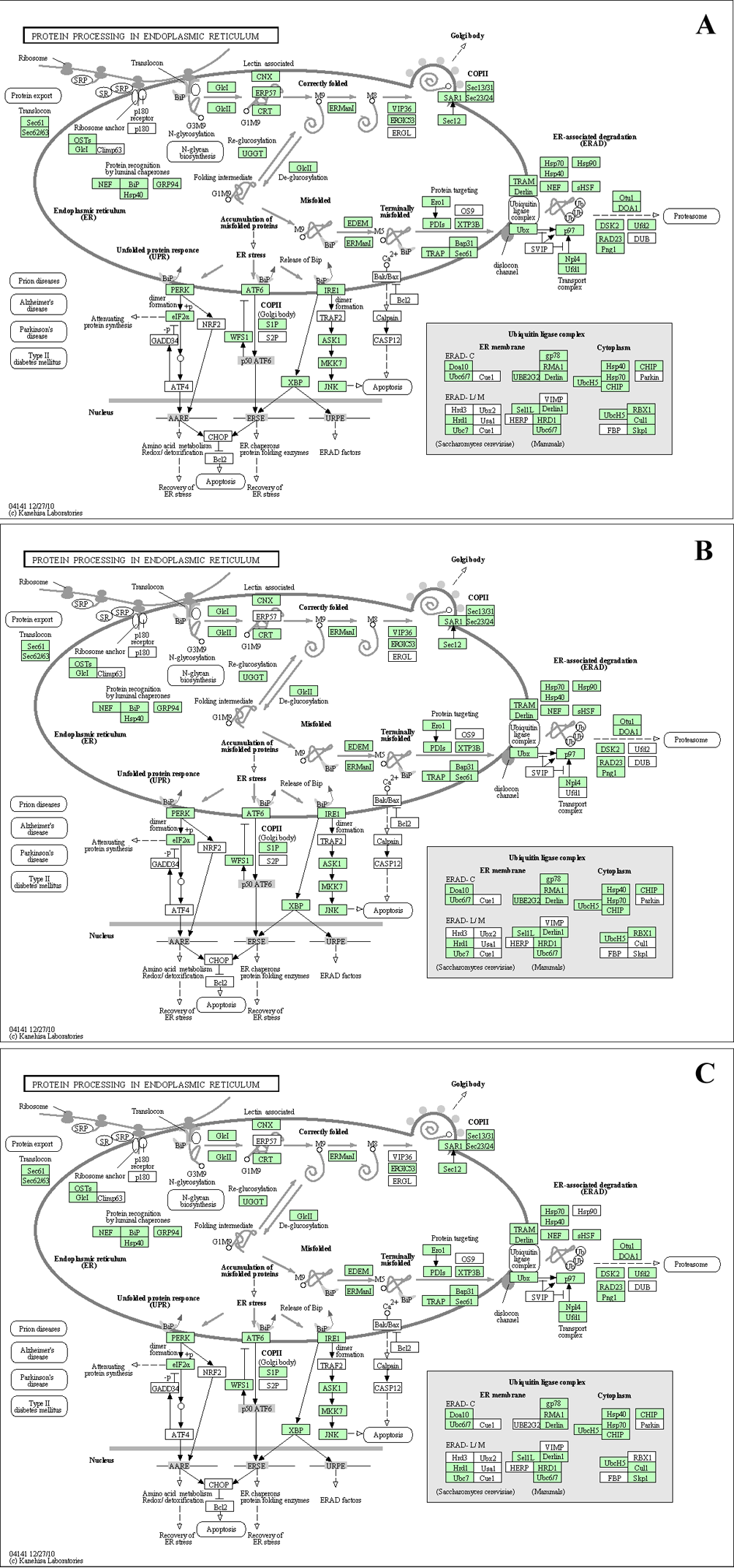

Supplement: Supplementary file 21 — Additional file 21: Figure S7: The oxidative phosphorylation pathway. (A) miRNA target genes detected in the entire period from the fourth instar molting to the fifth instar day 8 before spinning, (B) target genes first detected in this study; and (C) target genes detected in the third day of the fifth instar. Mapped pathways were highlighted in green. (TIFF 767 KB) [file 12864_2013_6094_MOESM21_ESM.tiff]

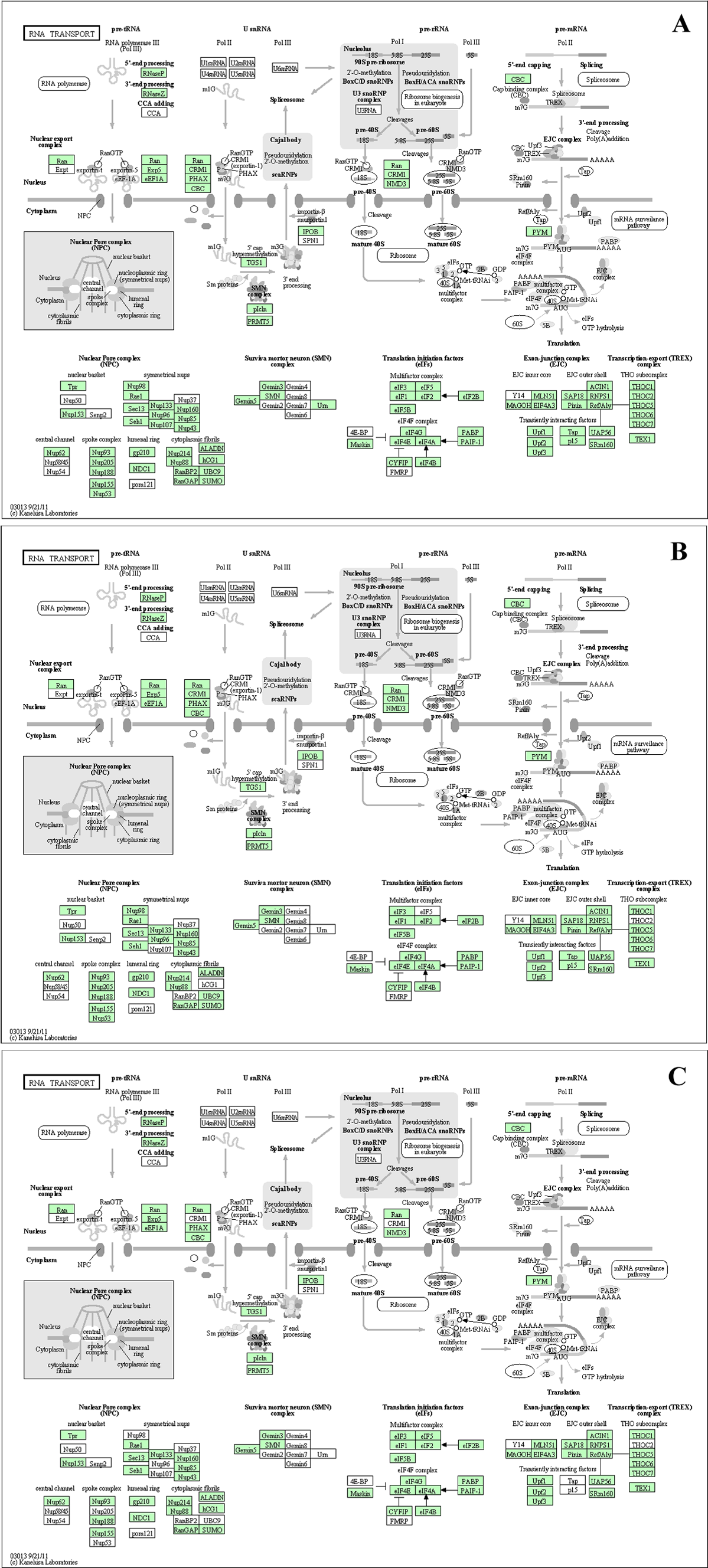

Supplement: Supplementary file 22 — Additional file 22: Figure S8: The purine metabolism pathway. (A) miRNA target genes detected in the entire period from the fourth instar molting to the fifth instar day 8 before spinning, (B) target genes first detected in this study; and (C) target genes detected in the third day of the fifth instar. Mapped pathways were highlighted in green. (TIFF 1 MB) [file 12864_2013_6094_MOESM22_ESM.tiff]

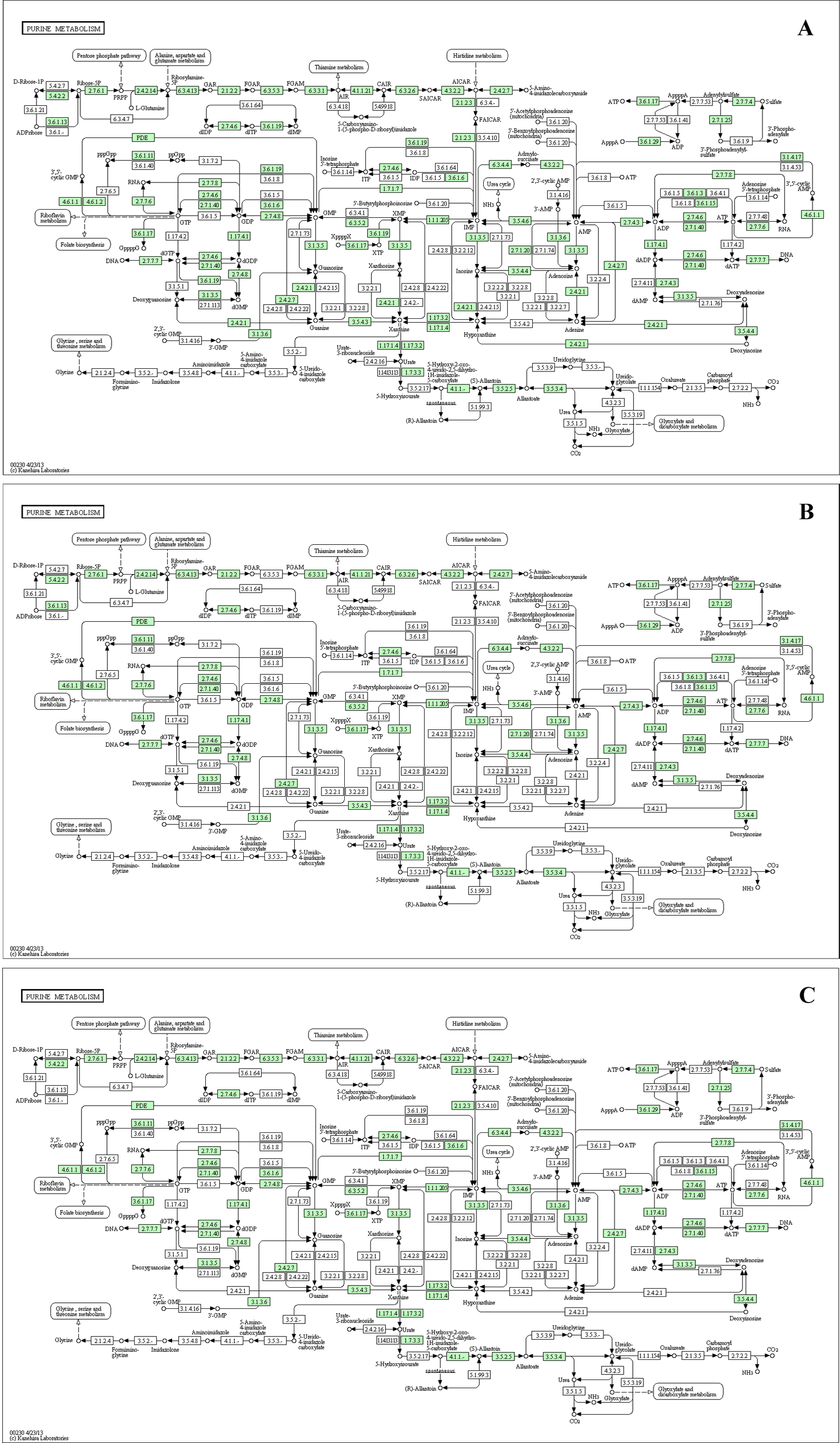

Supplement: Supplementary file 23 — Additional file 23: Figure S9: The pyrimidine metabolism pathway. (A) miRNA target genes detected in the entire period from the fourth instar molting to the fifth instar day 8 before spinning, (B) target genes first detected in this study; and (C) target genes detected in the third day of the fifth instar. Mapped pathways were highlighted in green. (TIFF 1 MB) [file 12864_2013_6094_MOESM23_ESM.tiff]

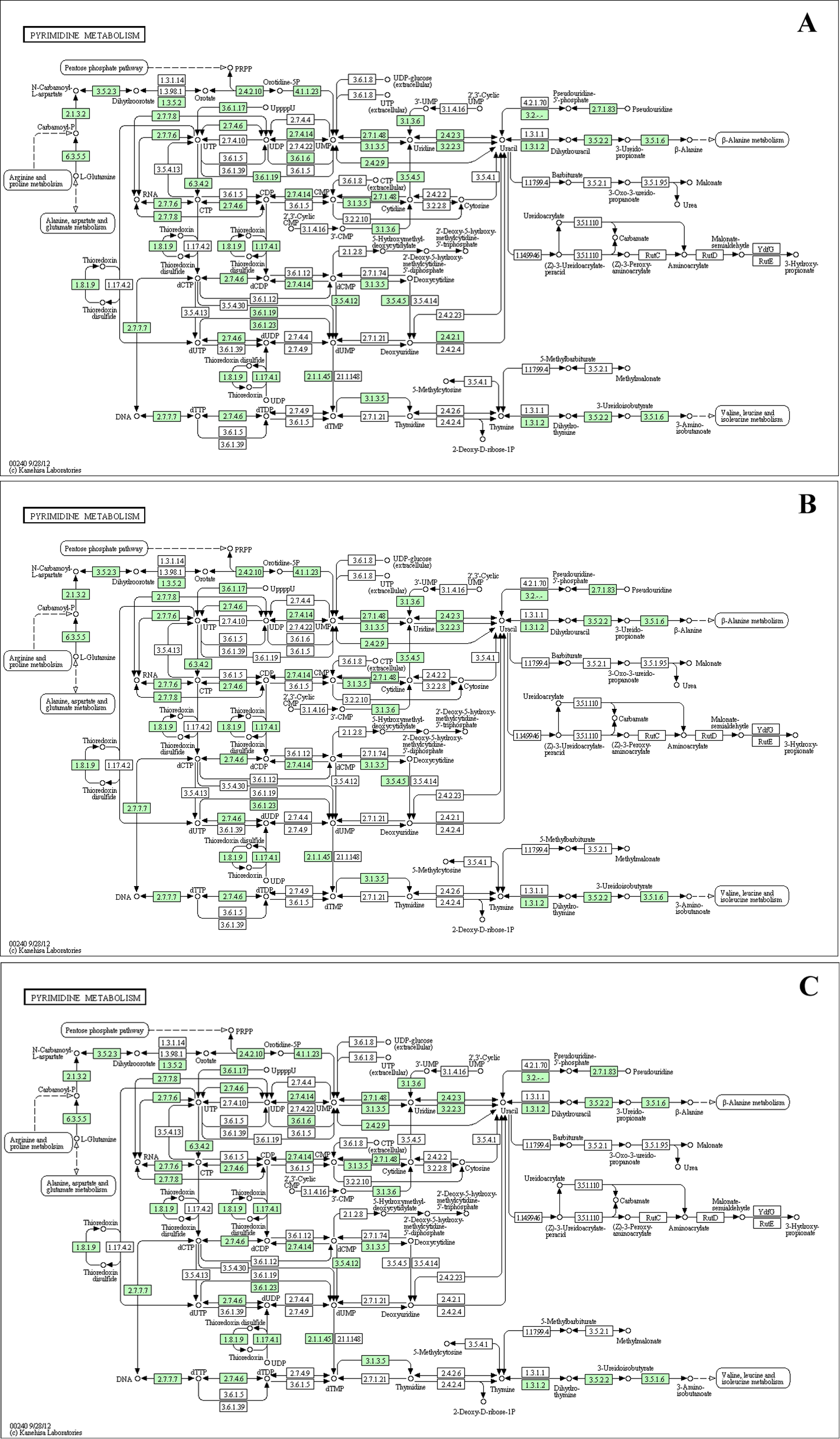

Supplement: Supplementary file 24 — Additional file 24: Figure S10: The RNA transport pathway. (A) miRNA target genes detected in the entire period from the fourth instar molting to the fifth instar day 8 before spinning, (B) target genes first detected in this study; and (C) target genes detected in the third day of the fifth instar. Mapped pathways were highlighted in green. (TIFF 1 MB) [file 12864_2013_6094_MOESM24_ESM.tiff]

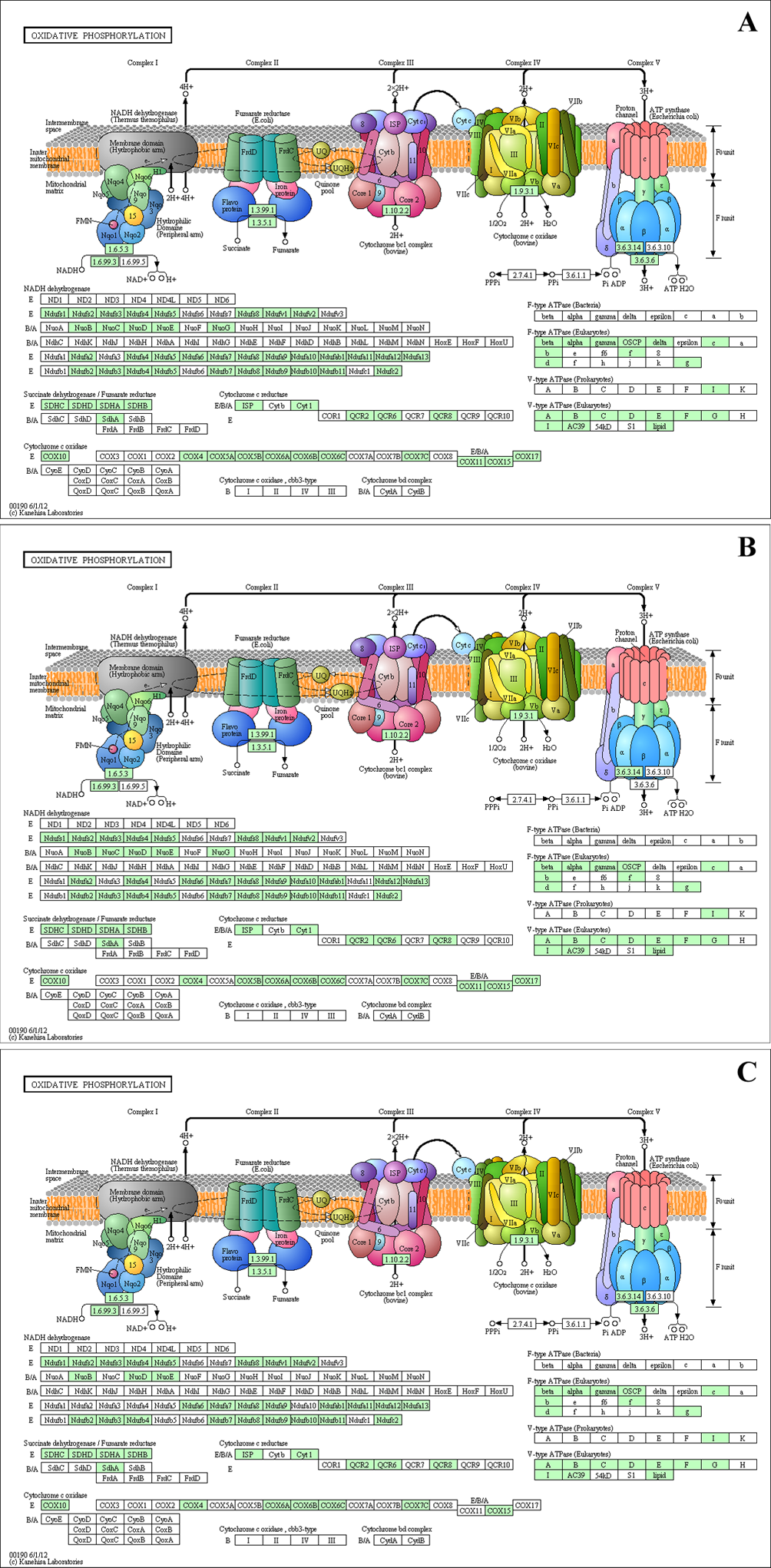

Supplement: Supplementary file 25 — Additional file 25: Figure S11: The cell cycle pathway. (A) miRNA target genes detected in the entire period from the fourth instar molting to the fifth instar day 8 before spinning, (B) target genes first detected in this study; and (C) target genes detected in the third day of the fifth instar. Mapped pathways were highlighted in green. (TIFF 1 MB) [file 12864_2013_6094_MOESM25_ESM.tiff]

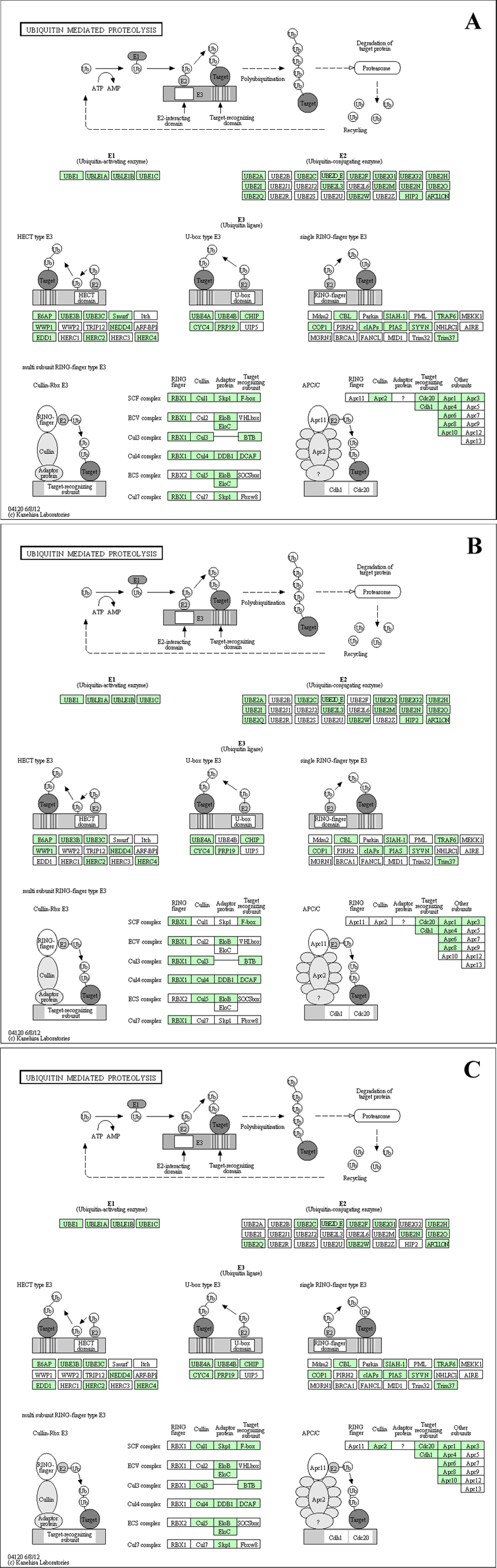

Supplement: Supplementary file 26 — Additional file 26: Figure S12: The ubiquitin mediated proteolysis pathway. (A) miRNA target genes detected in the entire period from the fourth instar molting to the fifth instar day 8 before spinning, (B) target genes first detected in this study; and (C) target genes detected in the third day of the fifth instar. Mapped pathways were highlighted in green. (TIFF 765 KB) [file 12864_2013_6094_MOESM26_ESM.tiff]

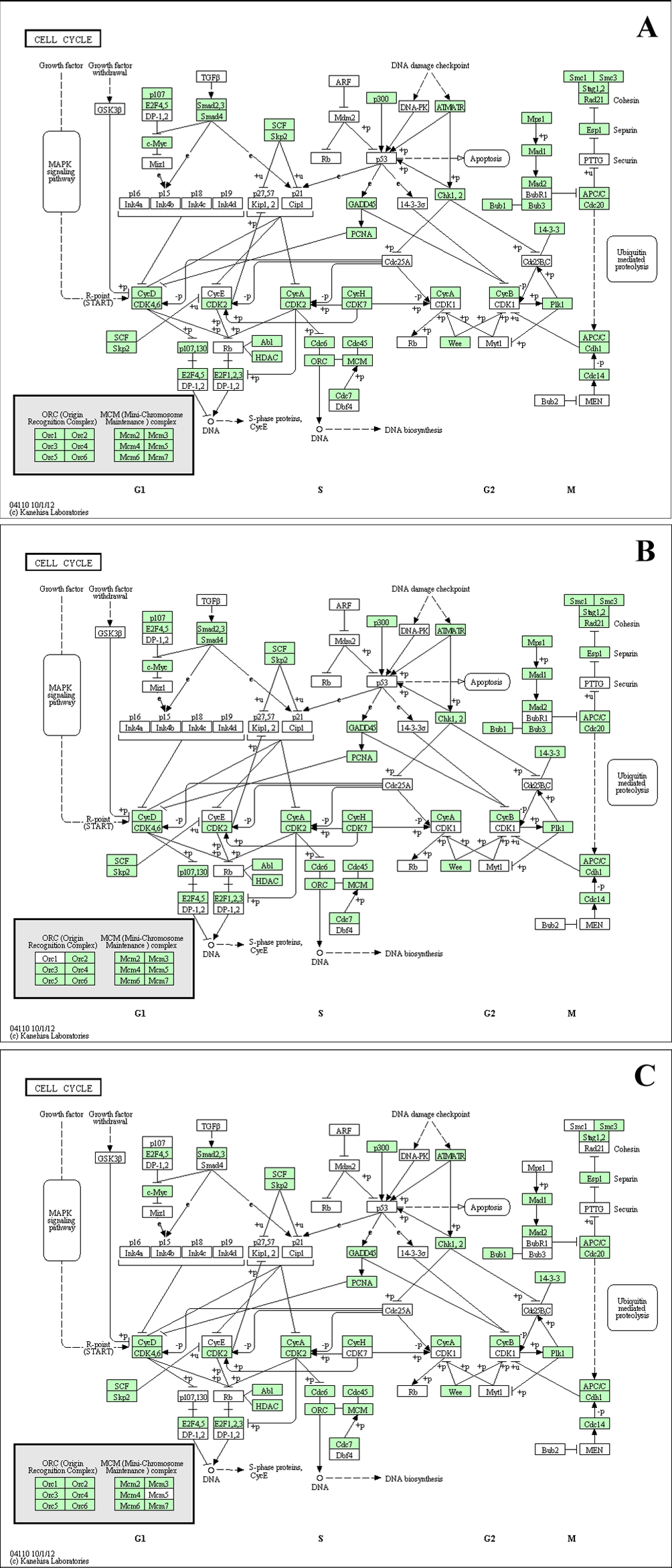

Supplement: Supplementary file 27 — Additional file 27: Figure S13: The protein processing in endoplasmic reticulum pathway. (A) miRNA target genes detected in the entire period from the fourth instar molting to the fifth instar day-8 before spinning, (B) target genes first detected in this study; and (C) target genes detected in the third day of the fifth instar. Mapped pathways were highlighted in green. (TIFF 757 KB) [file 12864_2013_6094_MOESM27_ESM.tiff]
