# Supplementary material for: MicroRNA expression profiling of the fifth-instar posterior silk gland of Bombyx mori
Source: BMC Genomics. 2014 May 29;15(1):410. doi: 10.1186/1471-2164-15-410 (PMC4045974; doi:10.1186/1471-2164-15-410)
Supplement: Supplementary file 10 — Additional file 10: Table S5: Significance testing for correlation of technical repeats by probes of repeated twice. (DOC 46 KB) [file 12864_2013_6094_MOESM10_ESM.doc]

**Supplemental table 5 Significance testing for correlation of technical repeats by probes of repeated twice**

| **Chip** | **Multiple R** | **R^2^** | **Adjusted R^2^** | **Standard error** | **Observed value** |
| --- | --- | --- | --- | --- | --- |
| **1** | 0.992986 | 0.9860206 | 0.986004 | 126.8527 | 841 |
| **2** | 0.997795 | 0.995595 | 0.99559 | 115.0989 | 841 |
| **3** | 0.998505 | 0.9970121 | 0.997009 | 82.4981 | 841 |
| **4** | 0.999123 | 0.9982464 | 0.998244 | 60.18148 | 841 |
| **5** | 0.999605 | 0.9992108 | 0.99921 | 34.39458 | 841 |
| **6** | 0.990344 | 0.9807804 | 0.980757 | 106.4798 | 841 |
| **7** | 0.996439 | 0.9928904 | 0.992882 | 157.8835 | 841 |
| **8** | 0.994538 | 0.9891051 | 0.989092 | 107.8021 | 841 |
| **9** | 0.995132 | 0.9902877 | 0.990276 | 116.3778 | 841 |
| **10** | 0.995575 | 0.9911692 | 0.991159 | 68.58641 | 841 |
| **11** | 0.997915 | 0.9958334 | 0.995828 | 66.70872 | 841 |
| **12** | 0.998727 | 0.9974564 | 0.997453 | 19.74329 | 841 |
| **13** | 0.996737 | 0.9934854 | 0.993478 | 92.15506 | 841 |
| **14** | 0.995079 | 0.9901817 | 0.99017 | 59.39007 | 841 |
| **15** | 0.997215 | 0.9944374 | 0.994431 | 60.45483 | 841 |
| **16** | 0.98894 | 0.9780016 | 0.977975 | 84.26884 | 841 |
